# MHC class I links with severe pathogenicity in C57BL/6N mice infected with SARS-CoV-2/BMA8

**DOI:** 10.1186/s12985-023-02031-0

**Published:** 2023-04-20

**Authors:** Tian Qin, Beilei Shen, Entao Li, Song Jin, Rongbo Luo, Yiming Zhang, Jing Qi, Xiuwen Deng, Zhuangzhuang Shi, Tiecheng Wang, Yifa Zhou, Yuwei Gao

**Affiliations:** 1 School of life sciences, Northeast Normal University, Changchun, 130024 China; 2 Changchun Veterinary Research Institute, Chinese Academy of Agricultural Sciences, Changchun, 130122 China; 3 Division of Life Sciences and Medicine, University of Science and Technology of China, Hefei, Anhui 230027 China; 4 College of life sciences, Shandong Normal University, Jinan, 250014 China; 5 College of Integrated Chinese and Western Medicine, Changchun University of Chinese Medicine, Changchun, Jilin 130117 China; 6 College of Animal Science and Technology, Jilin Agricultural University, Changchun, 130033 China

**Keywords:** SARS-CoV-2, Pathogenicity severity, MHC class I, CD8^+^ T cells, Immune response

## Abstract

**Background:**

The severe acute respiratory syndrome coronavirus 2 (SARS-CoV-2) causes non-symptomatic infection, mild influenza-like symptoms to pneumonia, severe acute respiratory distress syndrome, and even death, reflecting different clinical symptoms of viral infection. However, the mechanism of its pathogenicity remains unclear. Host-specific traits have a breakthrough significance for studying the pathogenicity of SARS-CoV-2. We previously reported SARS-CoV-2/BMA8, a mouse-adapted strain, was lethal to aged BALB/c mice but not to aged C57BL/6N mice. Here, we further investigate the differences in pathogenicity of BMA8 strain against wild-type aged C57BL/6N and BALB/c mice.

**Methods:**

Whole blood and tissues were collected from mice before and after BMA8 strain infection. Viral replication and infectivity were assessed by detection of viral RNA copies and viral titers; the degree of inflammation in mice was tested by whole blood cell count, ELISA and RT-qPCR assays; the pathogenicity of SARS-CoV-2/BMA8 in mice was measured by Histopathology and Immunohistochemistry; and the immune level of mice was evaluated by flow cytometry to detect the number of CD8^+^ T cells.

**Results:**

Our results suggest that SARS-CoV-2/BMA8 strain caused lower pathogenicity and inflammation level in C57BL/6N mice than in BALB/c mice. Interestingly, BALB/c mice whose MHC class I haplotype is H-2K^d^ showed more severe pathogenicity after infection with BMA8 strain, while blockade of H-2K^b^ in C57BL/6N mice was also able to cause this phenomenon. Furthermore, H-2K^b^ inhibition increased the expression of cytokines/chemokines and accelerated the decrease of CD8^+^ T cells caused by SARS-CoV-2/BMA8 infection.

**Conclusions:**

Taken together, our work shows that host MHC molecules play a crucial role in the pathogenicity differences of SARS-CoV-2/BMA8 infection. This provides a more profound insight into the pathogenesis of SARS-CoV-2, and contributes enlightenment and guidance for controlling the virus spread.

**Supplementary Information:**

The online version contains supplementary material available at 10.1186/s12985-023-02031-0.

## Background

Severe acute respiratory syndrome coronavirus 2 (SARS-CoV-2) is a highly transmissible pathogenic virus that seriously threatens human health and public safety. Similar to severe acute respiratory syndrome (SARS) and Middle East respiratory syndrome (MERS), the clinical manifestations of Coronavirus disease 2019 (COVID-19) include viral pneumonia, fever, cough and chest discomfort [1–3]. Some patients may also present with respiratory distress and bilateral lung infiltration, and even respiratory failure, systemic shock, or multi-organ failure [4, 5]. SARS-CoV-2 infection results in host immune dysfunction, such as lymphopenia, neutrophilia, dysregulation of monocytes and macrophages, reduced or delayed type I interferon responses and cytokine storm [6]. This suggests that the virus infection has host specificities, such as differential susceptibility to infection as well as differences in host immune responses.

Animal models have an important role in determining virus transmission routes, screening effective drugs, and evaluating vaccine development. Currently, the animal models commonly used for COVID-19 mainly include rhesus macaques [7], cats [8], ferrets [8], hamsters [9] and mice. The mouse model has a highly specific immune system and a rapid reproductive cycle [10]. SARS-CoV-2 enters host cells by binding to the cellular receptor angiotensin-converting enzyme 2 (ACE2); yet, standard laboratory mice do not support infection with SARS-CoV-2 because the virus S protein is incompatible with the mouse-human receptor homolog (mACE2) [11]. Recently, researchers have developed mouse models capable of being infected by SARS-CoV-2 through different strategies, including hACE2 transgenic mouse models [12], adenoviral vector transduction mouse models [13], and mouse-adapted strains of SARS-CoV-2 obtained by successive passages [14]. In addition, we established and validated a murine-adapted BMA8 lethal strain by introducing seven amine acid mutations (T819I, L1790F, I65S, Q498H, A22D, T67A and A36V) to a clinical isolate of SARS-CoV-2 (BetaCov/Wuhan/AMMS01/2020), which can reproduce the clinical manifestations of human COVID-19 in mice [15]. This strain can be used to explore the pathogenic mechanism and host inheritance-related to SARS-CoV-2. It also provides a convenient tool for evaluating COVID-19 vaccines and antiviral drugs.

In addition to ACE2, the major histocompatibility complex (MHC) is an important factor in host resistance to viral infection [16, 17]. The complex is a highly polymorphic gene cluster that is widely present in eukaryotic cells. When a virus infects cells, its antigens can be broken down into peptides that bind to MHC classes and are then expressed on the cell surface and subsequently recognized by CD4^+^ and CD8^+^ T cells [18]. Studies have shown that loss of T cells (CD4^+^ and CD8^+^) due to SARS-CoV-2 infection may lead to increased inflammatory response, whereas T-cell recovery may decrease inflammation during viral infection [19]. Furthermore, a clinical examination identified trends toward a decline in the number and quality of functional T cells in COVID-19 patients, which is strongly associated with disease severity and high mortality [19]. This suggests important roles of MHC-mediated T-cell responses during SARS-CoV-2 infection.

In this study, we showed differences in pathogenicity between C57BL/6N and BALB/c mice infected with SARS-CoV-2/BMA8 strain and revealed an important role of MHC-mediated T-cell responses in host pathogenicity.

## Methods

### Viruses, cell lines and mice

All virus infection experiments were performed in a biosafety level 3 laboratory (BSL-3). The mouse-adapted SARS-CoV-2 strain SARS-CoV-2/BMA8 (accession number: OL913103) originated from the Institute of Changchun Veterinary Research, Chinese Academy of Agricultural Sciences (Changchun, China) was used in this study [15]. The virus was passaged on African green monkey kidney epithelioid cells (Vero-E6) cultured in Dulbecco’s modified Eagle’s medium (DMEM; GIBCO, Grand Island, NY) supplemented with 10% fetal bovine serum (FBS; F8318, Sigma-Aldrich, USA) and 1% penicillin-streptomycin (30-002-CI, CORNING, USA) in a humidified atmosphere containing 5% CO_2_ at 37 ºC. 9-month-old specific pathogen-free (SPF) female mice (C57BL/6N and BALB/c) were purchased from Beijing Vital River Laboratory (Beijing, China).

### Animal experiments

All animals (mice) were inoculated intranasally with 50LD_50_ of the SARS-CoV-2/BMA8 strain after anesthetizing mice with isoflurane (R510-22, RWD, China). The body weight and living status of the mice were recorded every day. For MHC blocking experiments, H-2K^b^ mAbs (AF6-88.5.5.3, BioXCell, West Lebanon, NH) were used in C57BL/6N mice. The C57BL/6N mice were divided into 3 groups (n = 18 in each group): a control group (Control), a virus group (Virus), and an H-2K^b^ mAbs injection group (Virus + H-2K^b^). Two days before the virus infection, H-2K^b^ mAbs (2 mg/mL/day) was injected intraperitoneally for 5 consecutive days in the H-2K^b^ mAbs injection group.

### Complete blood cell counts

Whole blood was collected in sterile anticoagulation tubes and then analyzed by an automated haematology analyzer (BC-5000vet, Mindray, China) as described in the instructions. The analyzed parameters mainly included lymphocyte counts (Lym), neutrophil counts (Neu) and platelet counts (PLT).

### Extraction of RNA and quantitative real-time PCR

Total RNA from special tissues was extracted using the RNA simple Total RNA Kit (DP419, TIANGEN, China). The complementary DNA (cDNA) was used to perform experiments in a Real-Time PCR system (Bio-Rad, USA) using ChamQ Universal SYBR qPCR Master Mix (Q311, Vazyme, China). Mouse *Actb* was used as an internal control with 3 replicates per sample. The primer sequences are listed in (Additional file 1: Table S1). The expression of the SARS-CoV-2 *N* gene was detected by primers and probes to quantify viral RNA copies, the sequence was same as described in the previous study [15].

### Viral titers assay

Vero-E6 cells were seeded in 96-well plates and cultured in a cell incubator. After forming monolayer cells, the cells were used for virus titer analysis. The tissue homogenate supernatant was serially diluted 10-fold in DMEM and then seeded with 0.1 mL per well in monolayers of cells. After 1 h of incubation, the infected cells were cultured with DMEM supplemented with 2% FBS and 1% penicillin-streptomycin instead of medium. The cytopathological effect (CPE) was observed after continuing incubation 72 h, and the virus titer was calculated according to the Reed-Muench formula, expressed as the median tissue culture infectious dose (TCID_50_).

### Enzyme-linked immunosorbent assay (ELISA)

The serum samples from different groups of mice were collected according to the instructions, and the cytokine was measured using the V-PLEX Custom Mouse Cytokine 10-plex kit (K15048D, MSD, USA). The detected parameters included interferon (IFN-γ), interleukin (IL-1β, IL-2, IL-4, IL-5, IL-6, IL-10 and IL-12p70), keratinocyte chemoattractant/human growth-regulated oncogene (KC/GRO) and tumor necrosis factor-α (TNF-α). The data was collected by the MESO QuickPlex SQ120 plate reader (MSD, USA) and analyzed by Discovery Workbench software (v4.0, MSD, USA).

### Western blotting

Tissues were collected and grounded. Before quantitative determination, the whole proteins were extracted. The denatured protein samples were subsequently transferred to polyvinylidene difluoride (PVDF; Millipore, USA) membranes for immunoblotting assay. After using 5% bovine serum albumin (BSA; A1933, Sigma-Aldrich, USA) to block for 1 h at room temperature, membranes were incubated with anti-SARS-CoV-2 nucleocapsid protein (NP; 33717, CST, USA) at 4 ℃ overnight and then with secondary antibodies for 1 h at room temperature. Finally, the chemiluminescence imaging system (Tanon, China) was used for exposure. β-actin (ab8226, Abcam, UK) was used as a control.

### Peripheral blood mononuclear cell (PBMC) isolation and flow cytometry

Peripheral blood was collected into sterile anticoagulant tubes and mixed with red blood cell lysis buffer. The supernatant was then centrifuged (4000 rpm, 4 °C, 5 min) before abandonment. Cells were resuspended in 4% paraformaldehyde (PFA; P1110, Solarbio, China) diluted in phosphate-buffered saline (PBS; SH30256.01, HyClone, USA) and incubated for 10 min at room temperature. After centrifugation, the supernatant was removed and the cells were resuspended in PBS, and then cells could be safely removed from BSL-3. Subsequently, specific antibodies were incubated for 1 h at low temperature in the dark before testing on the BECKMAN CytoFLEX flowmeter (BECKMAN COULTER, USA), including CD3-APC (152306, BioLegend, USA), CD4-FITC (100510, BioLegend, USA) and CD8-PE (100708, BioLegend, USA). The visualized data were analyzed by using FlowJo V10 (Tree Star, Inc.) software.

### Histopathology and immunohistochemistry

Mice tissues (heart, liver, spleen, lung, kidney and intestine) were collected after euthanasia and fixed in 10% neutral buffered formalin (NBF). The ultrathin Sects. (4-5 μm) were prepared by paraffin embedding and stained with hematoxylin and eosin (H&E). The pathological score was determined by recording each section, and 1 point was added when cell necrosis, bleeding, wall thickening, inflammation or alveolar were observed. For immunohistochemistry (IHC), sections were incubated with SARS-CoV-2 nucleocapsid protein (vNP) antibody (CST, 33717, 1:200) for 30 min. The incubated sections were then labeled with horseradish peroxidase (HRP) and re-stained with hematoxylin before reacting with the chromogenic diaminobenzidine (DAB). After that, a transmission electron microscope (JEM1011, JEOL) was used for observation and recording.

### Statistical analysis

All experiments were performed in at least three independent replicates, and data were presented as mean ± SEM. Statistical analyses were performed using GraphPad Prism (version 8.0). Two-way ANOVA was used to analyze significant differences between C57BL/6N and BALB/c mice. P < 0.05 was considered statistically significant (*p < 0.05, **p < 0.01, ***p < 0.001, ns means no statistical difference).

## Results

### Characteristics of SARS-CoV-2/BMA8 infection in C57BL/6N and BALB/c mice

**Fig. 1:**
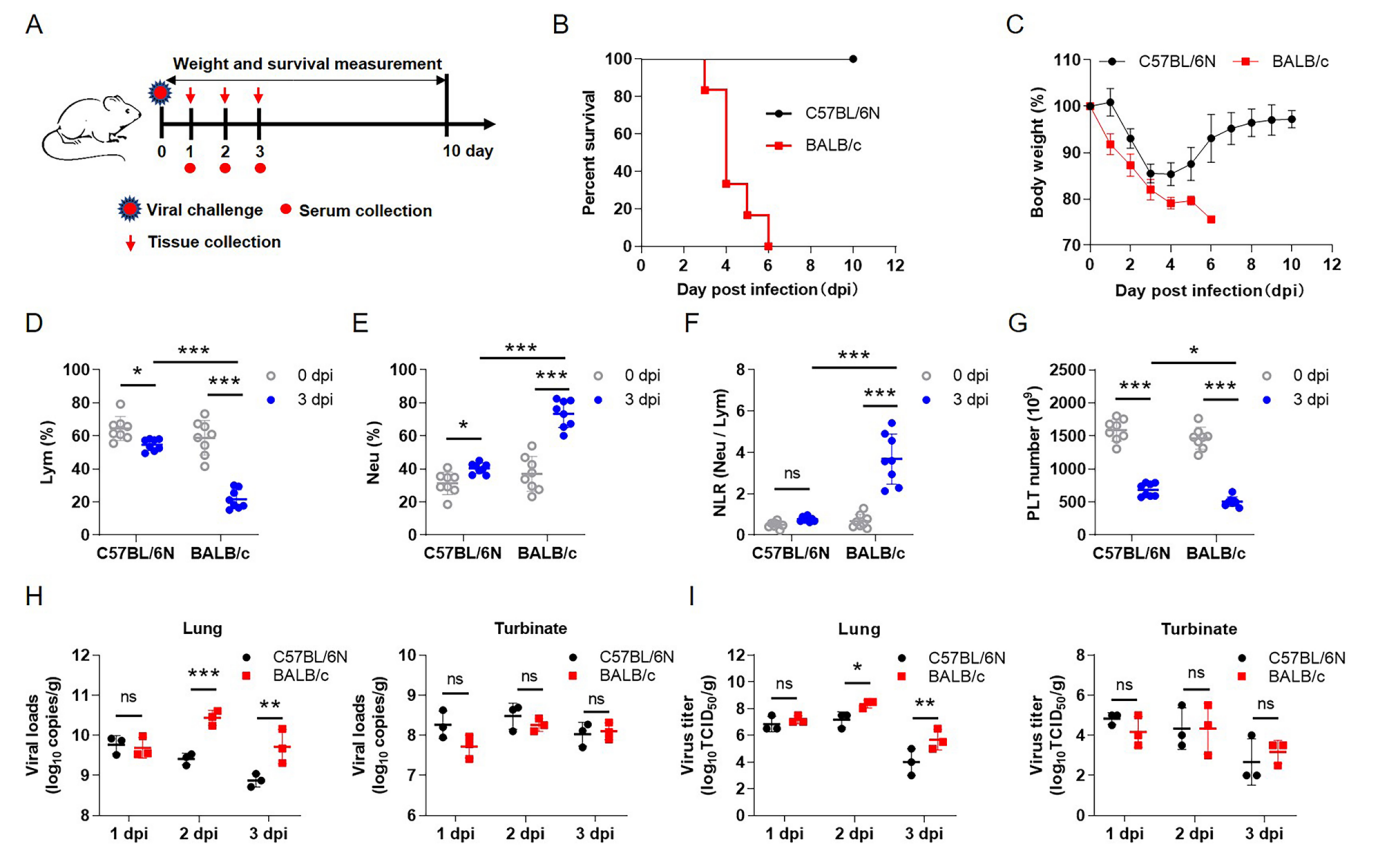
**Effects of BMA8 strain infection on C57BL/6N and BALB/c mice. (A)** The pattern of clinical surveillance in mice infected with BMA8 strain. **(B)** Survival curves of infected mice, n = 6. **(C)** Cumulative body weight analysis of infected mice. **(D-G)** The changes of parameters in the peripheral blood of mice analyzed at 0 dpi and 3 dpi; **(D)** percentage of lymphocytes (Lym), **(E)** percentage of neutrophils (Neu), **(F)** neutrophil-to-lymphocyte ratio (NLR), and **(G)** platelet count (PLT). **(H)** The viral RNA copies detected in lungs and turbinates of infected mice. **(I)** The virus titers were analyzed in lungs and turbinates of infected mice. *p < 0.05, **p < 0.01, ***p < 0.001, ns means no statistical difference

Previous studies have shown that the SARS-CoV-2 mouse-adapted strain SARS-CoV-2/BMA8 (BMA8) has different infectious properties in 9-month-old (aged) C57BL/6N and BALB/c mice [15]. i.e., this strain was lethal to BALB/c mice but not to C57BL/6N mice (Additional file 1: Fig. S1). The survival difference of different mouse strains infected with BMA8 provided us an ideal model to study the mechanism of host pathogenicity difference. To further investigate potential differences between the two types of mice infected with the BMA8 strain, clinical surveillance experiments were performed (Fig. 1A). We found that infection with BMA8 strain caused dramatic weight loss and, consequently, death in BALB/c mice but not in C57BL/6N mice (Fig. 1B, C), which is consistent with the previous research [15]. Additionally, the lymphocytes (Lym) and platelets (PLT) were decreased compared to the baseline value (before infection) (Fig. 1D, G), while neutrophils (Neu) were increased at 3 days post infection (dpi) (Fig. 1E); this phenomenon was more pronounced in BALB/c mice (Fig. 1F). Moreover, higher viral RNA copies (Fig. 1H) and viral titers (Fig. 1I) were detected in turbinates and lungs after BMA8 strain infection in C57BL/6N and BALB/c mice; both viral RNA copies and viral titers in lung tissues were significantly higher in BALB/c mice than in C57BL/6N mice. These findings indicate that BALB/c mice exhibit more significant susceptibility and lethality to the BMA8 strain than C57BL/6N mice.

### Pathogenicity of SARS-CoV-2/BMA8 against C57BL/6N and BALB/c mice

**Fig. 2:**
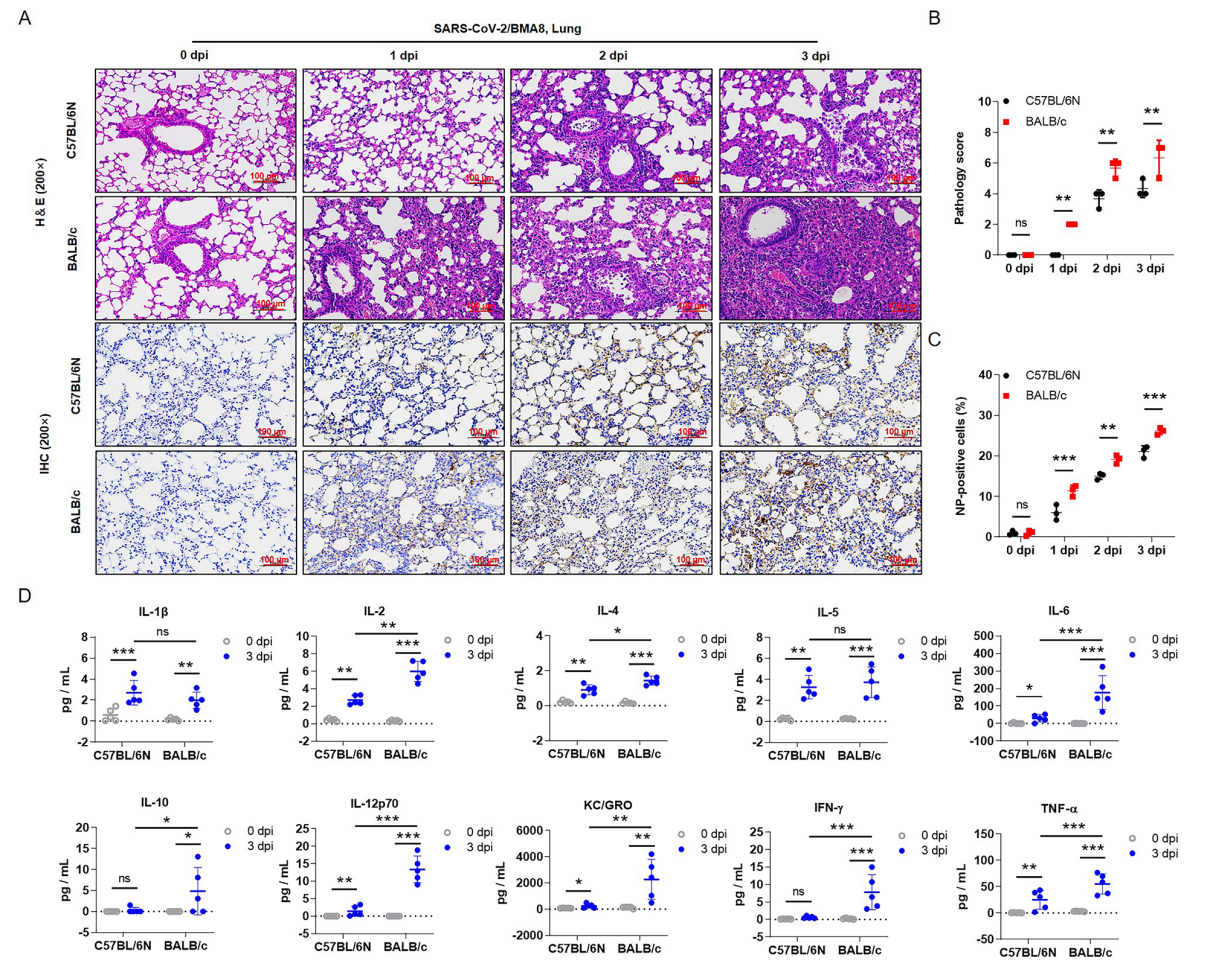
**Effect of BMA8 strain on pathogenicity of infected mice. (A)** H&E and immunohistochemical (IHC) staining were performed on the lung tissues of mice. **(B)** The pathology scores were summarized; 1 point was assigned for each alveolar wall thickening, hemorrhage, cell necrosis, and inflammation. **(C)** The percentage of NP-positive cells was summarized according to IHC staining in (A). **(D)** Cytokine concentrations were determined in mice serum at 0 dpi and 3 dpi, including IL-1β, IL-2, IL-4, IL-5, IL-6, IL-10, IL-12p70, KC/GRO, IFN-γ and TNF-α. *p < 0.05, **p < 0.01, ***p < 0.001, ns means no statistical difference

Next, the pathological examination of lung tissues from C57BL/6N and BALB/c mice revealed severe pathological alterations after BMA8 strain infection, including local bleeding, cell necrosis, alveolar wall thickening and inflammatory cell infiltration (Fig. 2A). In addition, the vNP-positive cells were also gradually increased in lung tissues of both types of mice; yet, higher pathology scores and vNP-positive cells appeared in BALB/c mice (Fig. 2B, C). This phenomenon was also observed in the heart, liver, spleen, kidney and intestine (Additional file 1: Fig. S2, S3). Subsequently, we detected the level of multiple inflammatory cytokines by ELISA assay to further compare the degree of inflammation in mice. The results showed that the protein concentration of inflammatory cytokines in both types of mice was significantly increased after BMA8 strain infection, and the expression of cytokines was higher in BALB/c mice, including IL-2, IL-4, IL-6, IL-10, IL-12p70, KC/GRO, IFN-γ and TNF-α (Fig. 2D). Furthermore, viral infection increased the mRNA expression of cytokines (*Il6*, *Tnf*), chemokines (*Cxcl10*, *Ccl2*), and interferon-responsive genes (*Ifnb1*, *Ifit3*, *Irf7*, *Gbp5* and *Isg15*) (Additional file 1: Fig. S4). These data collectively suggest that the BMA8 has stronger pathogenicity in BALB/c mice compared with C57BL/6N mice.

### The effect of MHC on C57BL/6N mice infected with SARS-CoV-2/BMA8 strain

**Fig. 3:**
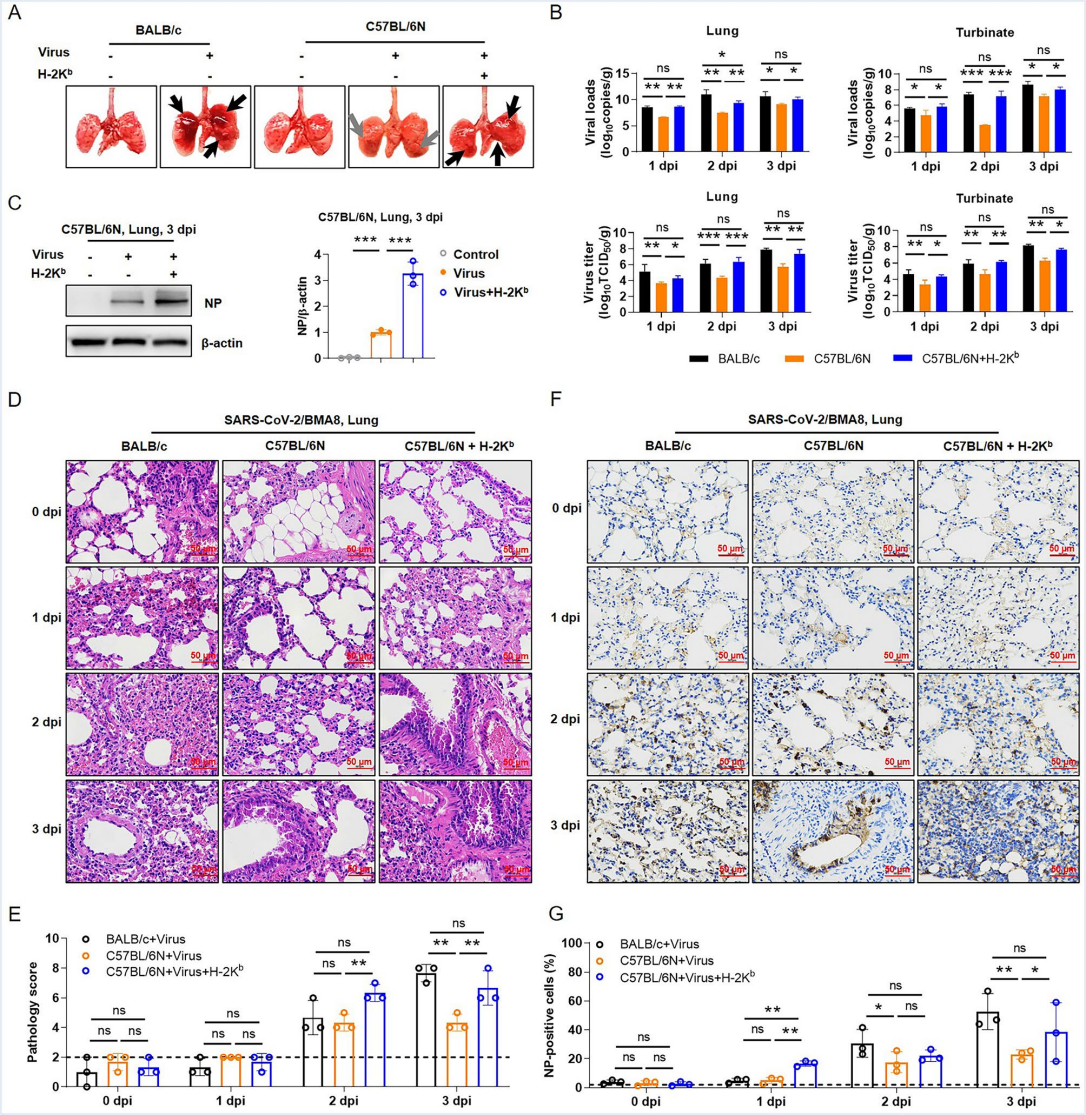
**The effect of H-2K**^**b**^**on pathogenicity of BMA8 strain in C57BL/6N mice. (A)** The lungs of different groups of mice were sampled and photographed, the gray arrows indicated mild bleeding, and the black arrow indicated severe tissue disease. **(B)** The viral RNA copies and viral titers were detected in lungs and turbinates of different groups. **(C)** The expression of vNP in lung tissues of BMA8-infected mice with or without H-2K^b^ mAbs treatment; β-actin was used as a loading control. **(D)** H&E staining was performed on the lung tissues of mice, showing representative photos of different groups. **(E)** The pathology scores were summarized according to Fig. 2B. **(F)** IHC analysis of NP expression in lung tissues. **(G)** The percentage of NP-positive cells was summarized according to IHC staining in (F). *p < 0.05, **p < 0.01, ***p < 0.001, ns means no statistical difference

Many studies have shown that differences in ACE2 receptor across species account for host differential susceptibility to SARS-CoV‐2 [8, 20, 21]. Therefore, we first extracted the *Ace2* genes from C57BL/6N and BALB/c mice and sequencing analysis showed that the *Ace2* gene sequences from C57BL/6N (Additional file 2) and BALB/c (Additional file 3) mice were perfectly aligned. And we detected the ACE2 protein expression levels in C57BL/6N and BALB/c mice by Western blot. The results show that ACE2 expression levels in lung and kidney tissues from C57BL/6N and BALB/c mice are identical (Additional file 1: Fig. S5). Then, the studies on the differences of host susceptibility caused by MHC molecules have attracted our attention. The MHC class I of C57BL/6N mice was H-2K^b^, whereas that of BALB/c mice was H-2K^d^ [22–24]. To investigate the roles of MHC in BMA8 strain infection, the C57BL/6N mice were treated with H-2K^b^ mAbs because the BMA8 strain was not lethal to the mice. Data on weight change and survival curve showed that BALB/c mice continued to lose weight until all died, but C57BL/6N mice did not, while the weight of C57BL/6N mice treated with H-2K^b^ mAbs decreased continuously before 7 dpi, and two mice died (Additional file 1: Fig. S6). And we found that the pathogenicity of the virus was enhanced in C57BL/6N mice after treatment with H-2K^b^ mAbs (Fig. 3A). Additionally, H-2K^b^ mAbs treatment significantly increased viral RNA copies and viral titers in the lung and turbinate of C57BL/6N-infected mice (Fig. 3B). Interestingly, the vNP was upregulated after H-2K^b^ mAbs treatment in lung tissues of SARS-CoV-2/BMA8-infected C57BL/6N mice (Fig. 3C). Moreover, H&E pathological analysis showed that the H-2K^b^ mAbs treatment resulted in the thickening of alveolar walls and infiltration of inflammatory cells in the lung tissues of infected mice (Fig. 3D). The H-2K^b^ mAbs treatment increased pathological damage caused by the BMA8 strain infection (Fig. 3E), and this phenomenon was also observed in the liver, spleen and kidney, except for the heart (Additional file 1: Fig. S7). We also found that the H-2K^b^ mAbs treatment significantly increased vNP expression in lung tissues (Fig. 3F, G), as well as heart, liver, spleen and kidney tissues (Additional file 1: Fig. S8).

To further compare the degree of inflammation in the infected mice before and after treatment with H-2K^b^ mAbs, the RT-qPCR assay was performed. The results showed that higher mRNA expression cytokines and chemokines were detected after H-2K^b^ mAbs treatment, including *Ifnb1*, *Cxcl9*, *Cxcl10*, *Ccl2*, *Ccl3*, *Ccl5*, *Il6*, *Tnf*, *Ifit3*, *Mx1* and *Irf7* (Additional file 1: Fig. S9). These findings indicated that inhibition of MHC could promote the pathogenicity of the BMA8 strain against C57BL/6N mice.

### Effect of MHC on the immune response of C57BL/6N mice infected with SARS-CoV-2/BMA8 strain

**Fig. 4:**
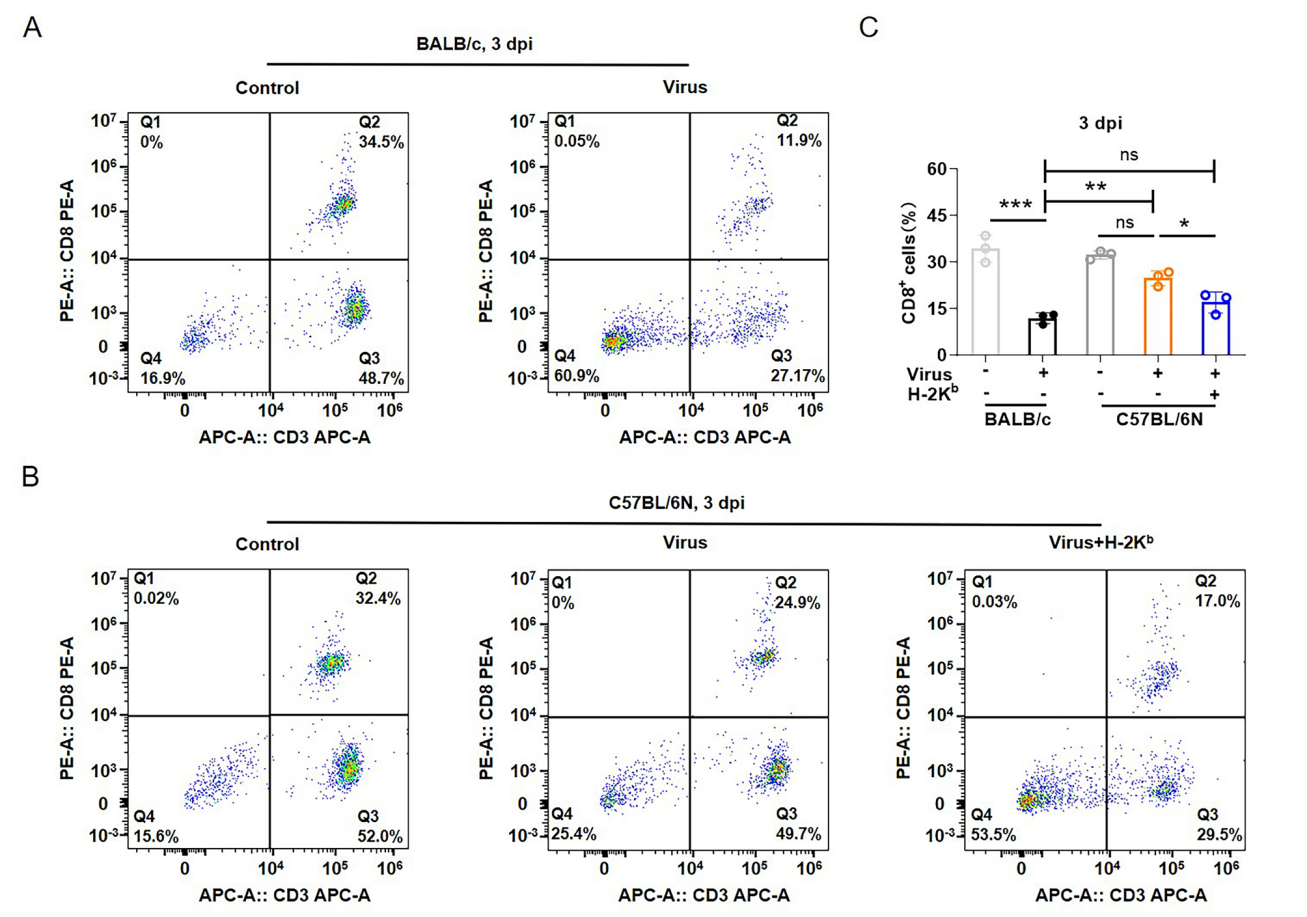
**Effect of BMA8 strain infection on the immune level in mice. (A)** CD8^+^ T cell amount changes in BALB/c mice infected with the BMA8 strain. **(B)** The changes of CD8^+^ T cell amount in C57BL/6N with or without H-2K^b^ mAbs treatment infected with the BMA8 strain. **(C)** Quantification of CD8^+^ T cells seen in (A, B). *p < 0.05, **p < 0.01, ***p < 0.001, ns means no statistical difference

To evaluate the innate immune response level of mice infected with SARS-CoV-2/BMA8 strain, the peripheral blood mononuclear cells (PBMCs) were collected at 3 dpi, and the CD8^+^ T cell number was detected by flow cytometry. In BALB/c mice, the number of CD8^+^ T cells was significantly decreased after infection with SARS-CoV-2/BMA8 (Fig. 4A, C). In addition, the H-2K^b^ mAbs treatment significantly reduced the amount of CD8^+^ T cells in C57BL/6N mice infected with SARS-CoV-2/BMA8 (Fig. 4B, C). These results suggest that inhibition of MHC decreases the strength of the host immune response during SARS-CoV-2/BMA8 infection and promotes viral replication *in vivo*.

## Discussion

Compared with other coronaviruses, SARS-CoV-2 is highly transmissible and pathogenic. The virus has a short production phase and a predominantly latent infection, despite expressing a limited number of proteins, some of which exert immunomodulatory roles in preventing recognition by the host immune surveillance system [25, 26]. For example, SARS-CoV-2 ORF6 blocks type II interferon-mediated immune responses and promote viral infection and replication [27]. In addition, angiotensin-converting enzyme 2 (ACE2) or major histocompatibility complex (MHC) alleles are associated with viral titers [20, 28]. MHC molecules are involved in antigen recognition and presentation in the immune response process. After the virus enters the body, MHC class I can deliver the peptide chain of the viral membrane to the outside of the cell for T cells to recognize and eliminate the virus [29]. Analysis of gene expression profiles of COVID-19 patients and SARS-CoV-2 infected epithelial cell lines showed that SARS-CoV-2 infection inhibits the activation of the MHC class I pathway, thereby facilitating viral escape from host immune surveillance [27]. The expression phenotype and genetic characteristics of host MHC molecules are closely related to viral infection. In this study, we examined differences in pathogenicity across mice infected with SARS-CoV-2/BMA8 and revealed an important role for MHC-mediated T-cell responses in host pathogenicity.

Notably, the MHC class I of C57BL/6N mice was H-2K^b^, whereas that of BALB/c was H-2K^d^. Therefore, we assume that the MHC class I/H-2K^b^ haplotype confers better immunity to C57BL/6N. Indeed, MHC inhibition promoted infection and pathogenicity of the SARS-CoV-2 mouse-adapted strain BMA8 in non-susceptible C57BL/6N mice. This also indicates the important role of MHC in the SARS-CoV-2 lifecycle and host immune response. Viral transmission to MHC class I mismatched individuals may result in the reversion of these mutations to restore viral fitness [30–32]. Therefore, we speculated that SARS-CoV-2 might evolve by selecting individual MHC class I-associated mutations and their reversion after multiple transmissions among individuals with highly diverse MHC class I haplotype. However, the specific mechanism needs to be further explored.

Inherent phenotypes of viruses and host characteristics are closely related to their pathogenicity, including differences in susceptibility and host immune responses [33]. The lymphocytes (T cells and B cells), which differentiate into specific effector cells by regulating cytokines and chemokines, are recruited to exert their biological effects at the site of infection initiation and vice versa [34]. T lymphocytes are important sources of many cytokines/chemokines and express various molecular receptors. For example, the CD4^+^ T cells differentiate into Th1 cells after stimulation with interleukin-12 (IL-12) and produce interferon-gamma (IFN-γ) to activate immune cells, thereby triggering response signaling and defense mechanisms against intracellular pathogens [35]. In the process of virus infection, CD8^+^ T cell-mediated immune responses are responsible for the clearance of viruses, such as influenza virus [36], respiratory syncytial virus [37] and severe acute respiratory syndrome coronavirus [38], and can even provide protection against secondary infections [39, 40]. Many studies also have shown that CD8^+^ T cell responses exert strong inhibitory pressure on SARS-CoV-2 replication [19, 33, 41]. Additionally, IL-6 induces the differentiation of CD8^+^ T cells to eliminate the virus by splitting the infected cells [42]. This suggests that the consumption of T cells may be an important reason for the decrease of lymphocytes in COVID-19 patients. Similarly, neutrophils and macrophages can be recruited to the injured area in the presence of chemokines, respectively, to recruit more immune cells to resist pathogens [43]. Clinical studies have shown that patients with COVID-19 often have thrombocytopenia syndrome, and platelets are activated to release downstream cytokines (or chemokines) to participate in immune regulation and response [44]. This is mainly because the complex relationship between cytokines (or chemokines) and their receptors enables them to be rapidly replenished in microenvironments, leading to the continued development of inflammatory response. Furthermore, inflammation and virus infection impact the function of platelets, causing clots to aggregate more rapidly, explaining increased thrombosis after SARS-CoV-2 infection [45]. Clinical studies have also found that lymphocytopenia and pro-inflammatory cytokine storm are more acute in COVID-19 severe patients than in mild patients and correlate with disease severity [46]. Consistently, our results also showed that the number of lymphocytes (especially CD8^+^ T cells) and platelets were significantly reduced after SARS-CoV-2 infection in a mouse model and that BALB/c mice with high cytokine expression had a higher degree of pathogenesis than C57BL/6N mice. Here, we detected the changes in the number of CD8^+^ T cells before and after infection, in order to better illustrate the potential mechanism of MHC affecting host pathogenicity, we also need to dynamically analyze the changes of systemic immune response (including NK cells, etc.) caused by the differences in host MHC molecules in future.

## Conclusion

In conclusion, by comparing the differences in pathogenicity of the SARS-CoV-2 mouse-adapted strains BMA8 in the two types of mice, we revealed the main roles of MHC molecules in mediating viral infection. In addition, we demonstrated the important function of CD8^+^ T cells in regulating the mice immune response against viral infection following SARS-CoV-2/BMA8 infection, which not only enriches the pathogenic mechanism of SARS-CoV-2, but also provides a novel strategy for the prevention and therapeutics of the virus.

## Electronic supplementary material

Below is the link to the electronic supplementary material.

Additional file 1 **Table****S1**. The list of RT-qPCR primers used in this study. **Fig.****S1**Acquisition of SARS-CoV-2 mouse-adapted strain BMA8. **Fig.****S2**The effects of BMA8 strain on pathogenicity of infected mice. **Fig.****S3** The NP expression in lung tissues of infected mice. **Fig. S4** The effects of BMA8 strain infection on the expression of inflammation-related genes in lung tissues of C57BL/6N and BALB/c mice at 0 dpi and 3 dpi, including *Ifnb1*, *Cxcl10*, *Ccl2*, *Il6*, *Tnf*, *Ifit3*, *Irf7*, *Gbp5* and *Isg15.* **Fig. S5** The expression levels of ACE2 protein in lung and kidney tissues of C57BL/6N and BALB/c mice; β-actin was used as a loading control. **Fig. S6** Effects of H-2K^b^ mAbs on body weight and survival rate of mice infected with BMA8 strain. **Fig. S7** Effects of H-2K^b^ mAbs on pathogenicity of BMA8 strain in C57BL/6N mice. **Fig. S8** The expression of NP in lung tissues of infected mice. **Fig. S9** The effects of BMA8 strain infection on the expression of inflammation-related genes in lung tissues of C57BL/6N and BALB/c mice with or without H-2K^b^ mAbs treatment, including *Ifnb1*, *Cxcl9*, *Cxcl10*, *Ccl2*, *Ccl3*, *Ccl5*, *Il6*, *Tnf*, *Ifit3*, *Mx1* and *Irf7*.

Additional file 2 The sequence of *Ace2* gene of C57BL/6N mice.

Additional file 3 The sequence of *Ace2* gene of BALB/c mice.

## Data Availability

All data generated or analyzed during this study are included in this published article and its supplementary information files.

## Abbreviations

SARS-CoV-2: Severe acute respiratory syndrome coronavirus 2
SARS: Severe acute respiratory syndrome
MERS: Middle East respiratory syndrome
COVID-19: Coronavirus disease 2019
ACE2: Angiotensin-converting enzyme 2
MHC: Major histocompatibility complex
BSL-3: Biosafety level 3 laboratory
Vero-E6: African green monkey kidney epithelioid cells
DMEM: Dulbecco’s modified Eagle’s medium
FBS: Fetal bovine serum
SPF: Specific pathogen-free
LD_50_: Median lethal dose
dpi: Days post infection
mAbs: Monoclonal antibodies
Lym: Lymphocyte
Neu: Neutrophil
NLR: Neutrophil to lymphocyte ratio
PLT: Platelet
cDNA: Complementary DNA
CPE: Cytopathological effect
TCID_50_: Median tissue culture infectious dose
RT-qPCR: Quantitative Real-time PCR
IFN-γ: Interferon-gamma
IL: Interleukin
KC/GRO: Keratinocyte chemoattractant/Human growth-regulated oncogene
TNF: Tumor necrosis factor
PVDF: Polyvinylidene difluoride
BSA: Bovine serum albumin
NP: Nucleocapsid protein
PBMC: Peripheral blood mononuclear cell
PFA: Paraformaldehyde
PBS: Phosphate-buffered saline
NBF: Neutral buffered formalin
H&E: Hematoxylin and eosin
IHC: Immunohistochemistry
HRP: Horseradish peroxidase
DAB: 3,3′-Diaminobenzidine
SEM: Standard error of mean
ANOVA: Analysis of variance
Cxcl9: C-X-C motif chemokine ligand 9
Cxcl10: C-X-C motif chemokine ligand 10
Ccl2: C-C motif chemokine ligand 2
Ccl3: C-C motif chemokine ligand 3
Ccl5: C-C motif chemokine ligand 5
Ifnb1: Interferon beta 1
Irf7: Interferon regulatory factor 7
Ifit3: Interferon induced protein with tetratricopeptide repeats 3
Isg15: ISG15 ubiquitin like modifier
Gbp5: Guanylate binding protein 5
Mx1: MX dynamin like GTPase 1
NK cells: Natural killer cells.

## References

[1] Jackson CB, Farzan M, Chen B, Choe H (2022). Mechanisms of SARS-CoV-2 entry into cells. Nat Rev Mol Cell Biol.

[2] Huang C, Wang Y, Li X, Ren L, Zhao J, Hu Y, Zhang L, Fan G, Xu J, Gu X (2020). Clinical features of patients infected with 2019 novel coronavirus in Wuhan, China. Lancet.

[3] V’Kovski P, Kratzel A, Steiner S, Stalder H, Thiel V (2021). Coronavirus biology and replication: implications for SARS-CoV-2. Nat Rev Microbiol.

[4] Guan WJ, Ni ZY, Hu Y, Liang WH, Ou CQ, He JX, Liu L, Shan H, Lei CL, Hui DSC (2020). Clinical characteristics of Coronavirus Disease 2019 in China. N Engl J Med.

[5] Chen N, Zhou M, Dong X, Qu J, Gong F, Han Y, Qiu Y, Wang J, Liu Y, Wei Y (2020). Epidemiological and clinical characteristics of 99 cases of 2019 novel coronavirus pneumonia in Wuhan, China: a descriptive study. Lancet.

[6] Yang L, Liu S, Liu J, Zhang Z, Wan X, Huang B, Chen Y, Zhang Y (2020). COVID-19: immunopathogenesis and immunotherapeutics. Signal Transduct Target Ther.

[7] Yu P, Qi F, Xu Y, Li F, Liu P, Liu J, Bao L, Deng W, Gao H, Xiang Z (2020). Age-related rhesus macaque models of COVID-19. Anim Model Exp Med.

[8] Shi J, Wen Z, Zhong G, Yang H, Wang C, Huang B, Liu R, He X, Shuai L, Sun Z (2020). Susceptibility of ferrets, cats, dogs, and other domesticated animals to SARS-coronavirus 2. Science.

[9] Chan JF, Zhang AJ, Yuan S, Poon VK, Chan CC, Lee AC, Chan WM, Fan Z, Tsoi HW, Wen L (2020). Simulation of the clinical and pathological manifestations of Coronavirus Disease 2019 (COVID-19) in a golden syrian Hamster model: implications for Disease Pathogenesis and Transmissibility. Clin Infect Dis.

[10] Cleary SJ, Pitchford SC, Amison RT, Carrington R, Robaina Cabrera CL, Magnen M, Looney MR, Gray E, Page CP (2020). Animal models of mechanisms of SARS-CoV-2 infection and COVID-19 pathology. Br J Pharmacol.

[11] Wan Y, Shang J, Graham R, Baric RS, Li F. Receptor Recognition by the Novel Coronavirus from Wuhan: an Analysis Based on Decade-Long Structural Studies of SARS Coronavirus.J Virol2020,94. 10.1128/JVI.00127-20 PMC7081895 31996437

[12] Jiang RD, Liu MQ, Chen Y, Shan C, Zhou YW, Shen XR, Li Q, Zhang L, Zhu Y, Si HR (2020). Pathogenesis of SARS-CoV-2 in transgenic mice expressing human angiotensin-converting enzyme 2. Cell.

[13] Sun J, Zhuang Z, Zheng J, Li K, Wong RL, Liu D, Huang J, He J, Zhu A, Zhao J (2020). Generation of a broadly useful model for COVID-19 pathogenesis, vaccination, and treatment. Cell.

[14] Gu H, Chen Q, Yang G, He L, Fan H, Deng YQ, Wang Y, Teng Y, Zhao Z, Cui Y (2020). Adaptation of SARS-CoV-2 in BALB/c mice for testing vaccine efficacy. Science.

[15] Yan F, Li E, Wang T, Li Y, Liu J, Wang W, Qin T, Su R, Pei H, Wang S (2022). Characterization of two heterogeneous Lethal mouse-adapted SARS-CoV-2 variants recapitulating Representative aspects of human COVID-19. Front Immunol.

[16] Shah VK, Firmal P, Alam A, Ganguly D, Chattopadhyay S. Overview of Immune Response During SARS-CoV-2 Infection: Lessons From the Past. Front Immunol 2020, 11:1949. 10.3389/fimmu.2020.01949 PMC7426442 32849654

[17] Harty JT, Tvinnereim AR, White DW (2000). CD8 + T cell effector mechanisms in resistance to infection. Annu Rev Immunol.

[18] Pishesha N, Harmand TJ, Ploegh HL. A guide to antigen processing and presentation.Nat Rev Immunol2022. 10.1038/s41577-022-00707-2 35418563

[19] Peng Y, Mentzer AJ, Liu G, Yao X, Yin Z, Dong D, Dejnirattisai W, Rostron T, Supasa P, Liu C (2020). Broad and strong memory CD4(+) and CD8(+) T cells induced by SARS-CoV-2 in UK convalescent individuals following COVID-19. Nat Immunol.

[20] Ren W, Zhu Y, Wang Y, Shi H, Yu Y, Hu G, Feng F, Zhao X, Lan J, Wu J (2021). Comparative analysis reveals the species-specific genetic determinants of ACE2 required for SARS-CoV-2 entry. PLoS Pathog.

[21] Zhao X, Chen D, Szabla R, Zheng M, Li G, Du P, Zheng S, Li X, Song C, Li R et al. Broad and Differential Animal Angiotensin-Converting Enzyme 2 Receptor Usage by SARS-CoV-2.J Virol2020,94. 10.1128/JVI.00940-20 PMC7459545 32661139

[22] Rawle FC, Knowles BB, Ricciardi RP, Brahmacheri V, Duerksen-Hughes P, Wold WS, Gooding LR (1991). Specificity of the mouse cytotoxic T lymphocyte response to adenovirus 5. E1A is immunodominant in H-2b, but not in H-2d or H-2k mice. J Immunol.

[23] Launois P, Pingel S, Himmelrich H, Locksley R, Louis J (2007). Different epitopes of the LACK protein are recognized by V beta 4 V alpha 8 CD4 + T cells in H-2b and H-2d mice susceptible to Leishmania major. Microbes Infect.

[24] Khanolkar A, Hartwig SM, Haag BA, Meyerholz DK, Harty JT, Varga SM (2009). Toll-like receptor 4 deficiency increases disease and mortality after mouse hepatitis virus type 1 infection of susceptible C3H mice. J Virol.

[25] Chan JF, Kok KH, Zhu Z, Chu H, To KK, Yuan S, Yuen KY (2020). Genomic characterization of the 2019 novel human-pathogenic coronavirus isolated from a patient with atypical pneumonia after visiting Wuhan. Emerg Microbes Infect.

[26] Zhang Y, Chen Y, Li Y, Huang F, Luo B, Yuan Y, Xia B, Ma X, Yang T, Yu F et al. The ORF8 protein of SARS-CoV-2 mediates immune evasion through down-regulating MHC-Ι.Proc Natl Acad Sci U S A2021,118. 10.1073/pnas.2024202118 PMC8201919 34021074

[27] Yoo JS, Sasaki M, Cho SX, Kasuga Y, Zhu B, Ouda R, Orba Y, de Figueiredo P, Sawa H, Kobayashi KS (2021). SARS-CoV-2 inhibits induction of the MHC class I pathway by targeting the STAT1-IRF1-NLRC5 axis. Nat Commun.

[28] Pishesha N, Harmand TJ, Rothlauf PW, Praest P, Alexander RK, van den Doel R, Liebeskind MJ, Vakaki MA, McCaul N, Wijne C et al. A class II MHC-targeted vaccine elicits immunity against SARS-CoV-2 and its variants.Proc Natl Acad Sci U S A2021,118. 10.1073/pnas.2116147118 PMC8612213 34654739

[29] Thimme R, Wieland S, Steiger C, Ghrayeb J, Reimann KA, Purcell RH, Chisari FV (2003). CD8(+) T cells mediate viral clearance and disease pathogenesis during acute hepatitis B virus infection. J Virol.

[30] Lin M, Tseng HK, Trejaut JA, Lee HL, Loo JH, Chu CC, Chen PJ, Su YW, Lim KH, Tsai ZU (2003). Association of HLA class I with severe acute respiratory syndrome coronavirus infection. BMC Med Genet.

[31] Ng MH, Lau KM, Li L, Cheng SH, Chan WY, Hui PK, Zee B, Leung CB, Sung JJ (2004). Association of human-leukocyte-antigen class I (B*0703) and class II (DRB1*0301) genotypes with susceptibility and resistance to the development of severe acute respiratory syndrome. J Infect Dis.

[32] Castelli EC, de Castro MV, Naslavsky MS, Scliar MO, Silva NSB, Andrade HS, Souza AS, Pereira RN, Castro CFB, Mendes-Junior CT (2021). MHC variants Associated with Symptomatic Versus Asymptomatic SARS-CoV-2 infection in highly exposed individuals. Front Immunol.

[33] Rydyznski Moderbacher C, Ramirez SI, Dan JM, Grifoni A, Hastie KM, Weiskopf D, Belanger S, Abbott RK, Kim C, Choi J (2020). Antigen-Specific Adaptive immunity to SARS-CoV-2 in Acute COVID-19 and Associations with Age and Disease Severity. Cell.

[34] Tan Y, Tang F (2021). SARS-CoV-2-mediated immune system activation and potential application in immunotherapy. Med Res Rev.

[35] Krueger PD, Goldberg MF, Hong SW, Osum KC, Langlois RA, Kotov DI, Dileepan T, Jenkins MK (2021). Two sequential activation modules control the differentiation of protective T helper-1 (Th1) cells. Immunity.

[36] Slütter B, Pewe LL, Kaech SM, Harty JT (2013). Lung airway-surveilling CXCR3(hi) memory CD8(+) T cells are critical for protection against influenza a virus. Immunity.

[37] Graham BS, Bunton LA, Wright PF, Karzon DT (1991). Role of T lymphocyte subsets in the pathogenesis of primary infection and rechallenge with respiratory syncytial virus in mice. J Clin Invest.

[38] Channappanavar R, Fett C, Zhao J, Meyerholz DK, Perlman S (2014). Virus-specific memory CD8 T cells provide substantial protection from lethal severe acute respiratory syndrome coronavirus infection. J Virol.

[39] Kinnear E, Lambert L, McDonald JU, Cheeseman HM, Caproni LJ, Tregoning JS (2018). Airway T cells protect against RSV infection in the absence of antibody. Mucosal Immunol.

[40] McMaster SR, Wilson JJ, Wang H, Kohlmeier JE (2015). Airway-Resident memory CD8 T cells provide Antigen-Specific Protection against Respiratory Virus Challenge through Rapid IFN-γ production. J Immunol.

[41] Sette A, Crotty S (2021). Adaptive immunity to SARS-CoV-2 and COVID-19. Cell.

[42] Copaescu A, Smibert O, Gibson A, Phillips EJ, Trubiano JA (2020). The role of IL-6 and other mediators in the cytokine storm associated with SARS-CoV-2 infection. J Allergy Clin Immunol.

[43] Merad M, Blish CA, Sallusto F, Iwasaki A (2022). The immunology and immunopathology of COVID-19. Science.

[44] Barrett TJ, Cornwell M, Myndzar K, Rolling CC, Xia Y, Drenkova K, Biebuyck A, Fields AT, Tawil M, Luttrell-Williams E (2021). Platelets amplify endotheliopathy in COVID-19. Sci Adv.

[45] Shen S, Zhang J, Fang Y, Lu S, Wu J, Zheng X, Deng F (2021). SARS-CoV-2 interacts with platelets and megakaryocytes via ACE2-independent mechanism. J Hematol Oncol.

[46] Liu J, Li S, Liu J, Liang B, Wang X, Wang H, Li W, Tong Q, Yi J, Zhao L (2020). Longitudinal characteristics of lymphocyte responses and cytokine profiles in the peripheral blood of SARS-CoV-2 infected patients. EBioMedicine.

