# Supplementary material for: MHC class I links with severe pathogenicity in C57BL/6N mice infected with SARS-CoV-2/BMA8
Source: Virol J. 2023 Apr 20;20:75. doi: 10.1186/s12985-023-02031-0 (PMC10116088; doi:10.1186/s12985-023-02031-0)
Supplement: Supplementary file 1 — Additional file 1 TableS1. The list of RT-qPCR primers used in this study. Fig.S1Acquisition of SARS-CoV-2 mouse-adapted strain BMA8. Fig.S2The effects of BMA8 strain on pathogenicity of infected mice. Fig.S3 The NP expression in lung tissues of infected mice. Fig. S4 The effects of BMA8 strain infection on the expression of inflammation-related genes in lung tissues of C57BL/6N and BALB/c mice at 0 dpi and 3 dpi, including Ifnb1, Cxcl10, Ccl2, Il6, Tnf, Ifit3, Irf7, Gbp5 and Isg15. Fig. S5 The expression levels of ACE2 protein in lung and kidney tissues of C57BL/6N and BALB/c mice; β-actin was used as a loading control. Fig. S6 Effects of H-2Kb mAbs on body weight and survival rate of mice infected with BMA8 strain. Fig. S7 Effects of H-2Kb mAbs on pathogenicity of BMA8 strain in C57BL/6N mice. Fig. S8 The expression of NP in lung tissues of infected mice. Fig. S9 The effects of BMA8 strain infection on the expression of inflammation-related genes in lung tissues of C57BL/6N and BALB/c mice with or without H-2Kb mAbs treatment, including Ifnb1, Cxcl9, Cxcl10, Ccl2, Ccl3, Ccl5, Il6, Tnf, Ifit3, Mx1 and Irf7. [file 12985_2023_2031_MOESM1_ESM.docx]

**Supplementary Information for**

**MHC class Ⅰ links with severe pathogenicity in C57BL/6N mice infected with SARS-CoV-2/BMA8**

Tian Qin^1,2^, Beilei Shen^2^, Entao Li^3^, Song Jin^2,4^, Rongbo Luo^2^, Yiming Zhang^2^, Jing Qi^1,2^, Xiuwen Deng^2,5^, Zhuangzhuang Shi^2,6^, Tiecheng Wang^2^, Yifa Zhou^1*^ and Yuwei Gao^2*^

^1^ School of life sciences, Northeast Normal University, Changchun 130024, China.

^2^ Changchun Veterinary Research Institute, Chinese Academy of Agricultural Sciences. Changchun 130122, China.

^3^ Division of Life Sciences and Medicine, University of Science and Technology of China, Hefei, Anhui 230027, China.

^4^ College of life sciences, Shandong Normal University, Jinan 250014, China.

^5^ College of Integrated Chinese and Western Medicine, Changchun University of Chinese Medicine, Changchun, Jilin, 130117, China.

^6^ College of Animal Science and Technology, Jilin Agricultural University, Changchun 130033, China.

****Corresponding author:*** Y, F. Zhou, School of life sciences, Northeast Normal University, Changchun 130024, China. [zhouyf383@nenu.edu.cn](mailto:zhouyf383@nenu.edu.cn); Y, W. Gao, Changchun Veterinary Research Institute, Chinese Academy of Agricultural Sciences. Changchun, Jilin 130122, China. [gaoyuwei@gmail.com](mailto:gaoyuwei@gmail.com)

***Running title:*** Host barrier of SARS-CoV-2 in mice

**Contents:**

**Table S1.** The list of RT-qPCR primers used in this study.

**Fig. S1** Acquisition of SARS-CoV-2 mouse-adapted strain BMA8.

**Fig. S2** The effects of BMA8 strain on pathogenicity of infected mice.

**Fig. S3** The NP expression in lung tissues of infected mice.

**Fig. S4** The effects of BMA8 strain infection on the expression of inflammation-related genes in lung tissues of C57BL/6N and BALB/c mice at 0 dpi and 3 dpi, including *Ifnb1*, *Cxcl10*, *Ccl2*, *Il6*, *Tnf*, *Ifit3*, *Irf7*, *Gbp5* and *Isg15.*

**Fig. S5** The expression levels of ACE2 protein in lung and kidney tissues of C57BL/6N and BALB/c mice; β-actin was used as a loading control.

**Fig. S6** Effects of H-2K^b^ mAbs on body weight and survival rate of mice infected with BMA8 strain.

**Fig. S7** Effects of H-2K^b^ mAbs on pathogenicity of BMA8 strain in C57BL/6N mice.

**Fig. S8** The expression of NP in lung tissues of infected mice.

**Fig. S9** The effects of BMA8 strain infection on the expression of inflammation-related genes in lung tissues of C57BL/6N and BALB/c mice with or without H-2K^b^ mAbs treatment, including *Ifnb1*, *Cxcl9*, *Cxcl10*, *Ccl2*, *Ccl3*, *Ccl5*, *Il6*, *Tnf*, *Ifit3*, *Mx1* and *Irf7.*

**Table S1. The list of RT-qPCR primers used in this study.**

| **Primer name** | **Primer sequence (5’ to 3’)** |
| --- | --- |
| *Actb* | F: 5’-TGAGCTGCGTTTTACACCCT-3’ |
|  | R: 5’-GCCTTCACCGTTCCAGTTTT-3’ |
| *Ifnb1* | F: 5’-GTCCTCAACTGCTCTCCACT-3’ |
|  | R: 5’-CCTGCAACCACCACTCATTC-3’ |
| *Il6* | F: 5’-ACAAGTCGGAGGCTTAATTACACAT-3’ |
|  | R: 5’-TTGCCATTGCACAACTCTTTTC-3’ |
| *Tnf* | F: 5’-ATGTCTCAGCCTCTTCTCATTC-3’ |
|  | R: 5’-GCTTGTCACTCGAATTTTGAGA-3’ |
| *Ccl2* | F: 5’-TTTTTGTCACCAAGCTCAAGAG-3’ |
|  | R: 5’-TTCTGATCTCATTTGGTTCCGA-3’ |
| *Ccl3* | F: 5’-CATATGGAGCTGACACCCCG-3’ |
|  | R: 5’-TTGGAGTCAGCGCAGATCTG-3’ |
| *Ccl5* | F: 5’-GACACCACTCCCTGCTGCTTTG-3’ |
|  | R: 5’-CTCTGGGTTGGCACACACTTGG-3’ |
| *Cxcl9* | F: 5’-CGAGGCACGATCCACTACAA-3’ |
|  | R: 5’-AGGCAGGTTTGATCTCCGTT-3’ |
| *Cxcl10* | F: 5’-ATCATCCCTGCGAGCCTATCCT-3’ |
|  | R: 5’-GACCTTTTTTGGCTAAACGCTTTC-3’ |
| *Ifit3* | F: 5’-CTCAGAACCAGTACCTGAAAGT-3’ |
|  | R: 5’-TTTCTTGTAAAACTGAGCTGCC-3’ |
| *Irf7* | F: 5’-AAATAGGGAAGAAGTGAGCCTC-3’ |
|  | R: 5’-CCCTTGTACATGATGGTCACAT-3’ |
| *Isg15* | F: 5’-CGATTTCCTGGTGTCCGTGACTAAC-3’ |
|  | R: 5’-TAAGACCGTCCTGGAGCACTGC-3’ |
| *Gbp5* | F: 5’-TTGGTTCTGCTTGACACTGAAGGC-3’ |
|  | R: 5’-AGGTGCTACTGAGGAGGATTGCTAG-3’ |
| *Mx1* | F: 5’-AAGGTCTTGGATGTGATGCGGAAC-3’ |
|  | R: 5’-GCTGCTCTTGGATGTCCTGCTG-3’ |


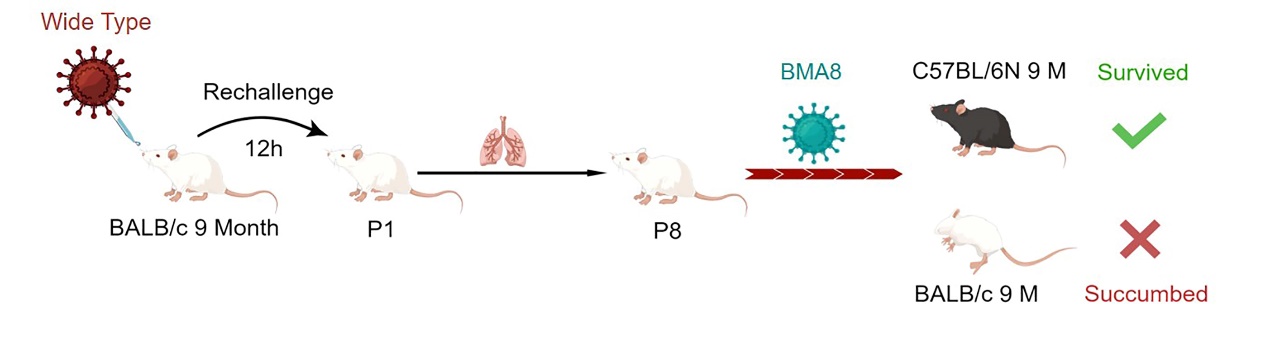


**Fig. S1 Acquisition of SARS-CoV-2 mouse-adapted strain BMA8.** The human clinical isolates of SARS-CoV-2 (Beta-Cov/Wuhan/AMMS01/2020) were continued serial passage in the lungs of 9-month-old BALB/c mice. After 8 repetitions, the mouse-adapted strain (SARS-CoV-2/BMA8, abbreviated as BMA8) was obtained, which was lethal to BALB/c but not to C57BL/6N mice. This graph was generated by figdraw ([www.figdraw.com](http://www.figdraw.com/)).

**
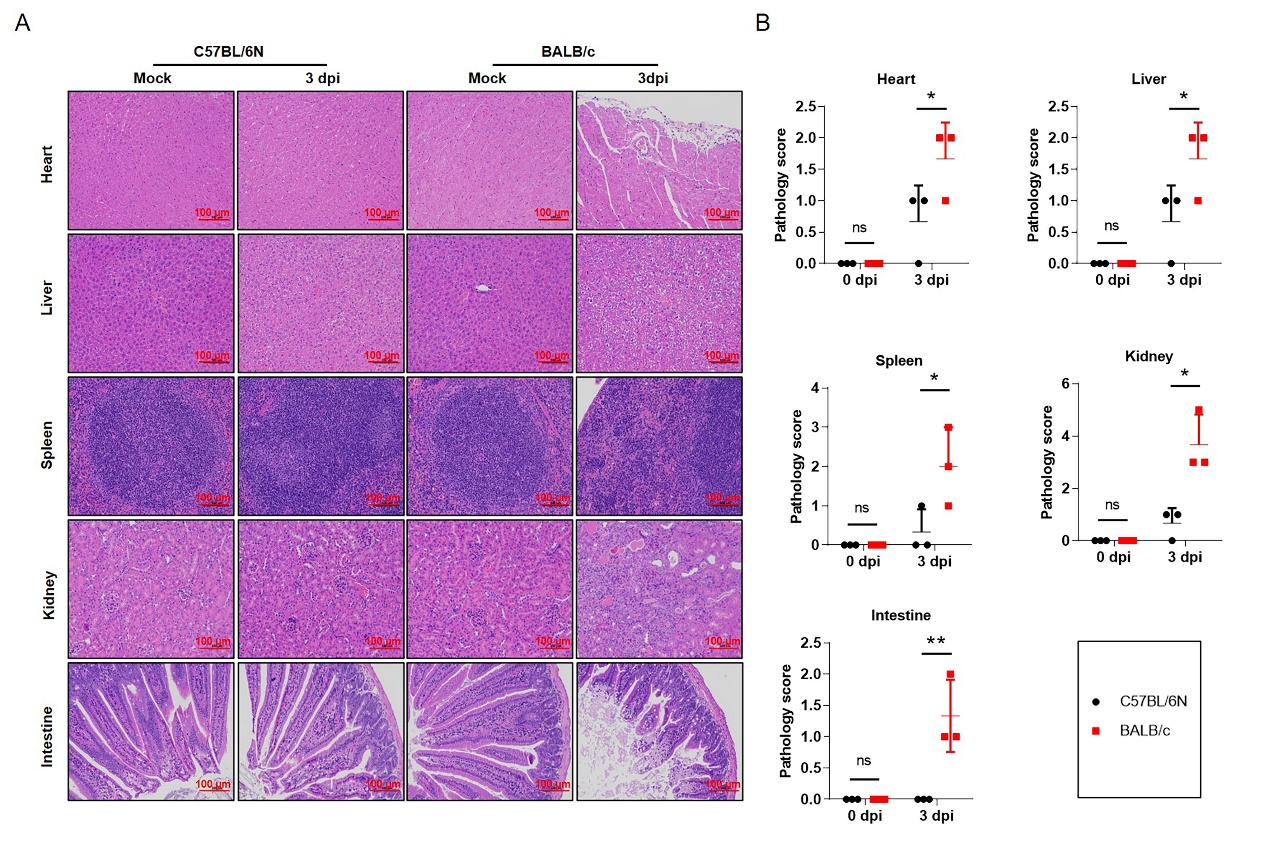
**

**Fig. S2 The effects of BMA8 strain on pathogenicity of infected mice. (A)** H&E staining was performed on the tissues of mice at 0 dpi and 3 dpi, showing representative photos of different groups, including heart, liver, spleen, kidney and intestinal tissues. **(B)** The pathology scores were summarized according to Fig. 2B. *p < 0.05, **p < 0.01, ns means no statistical difference.


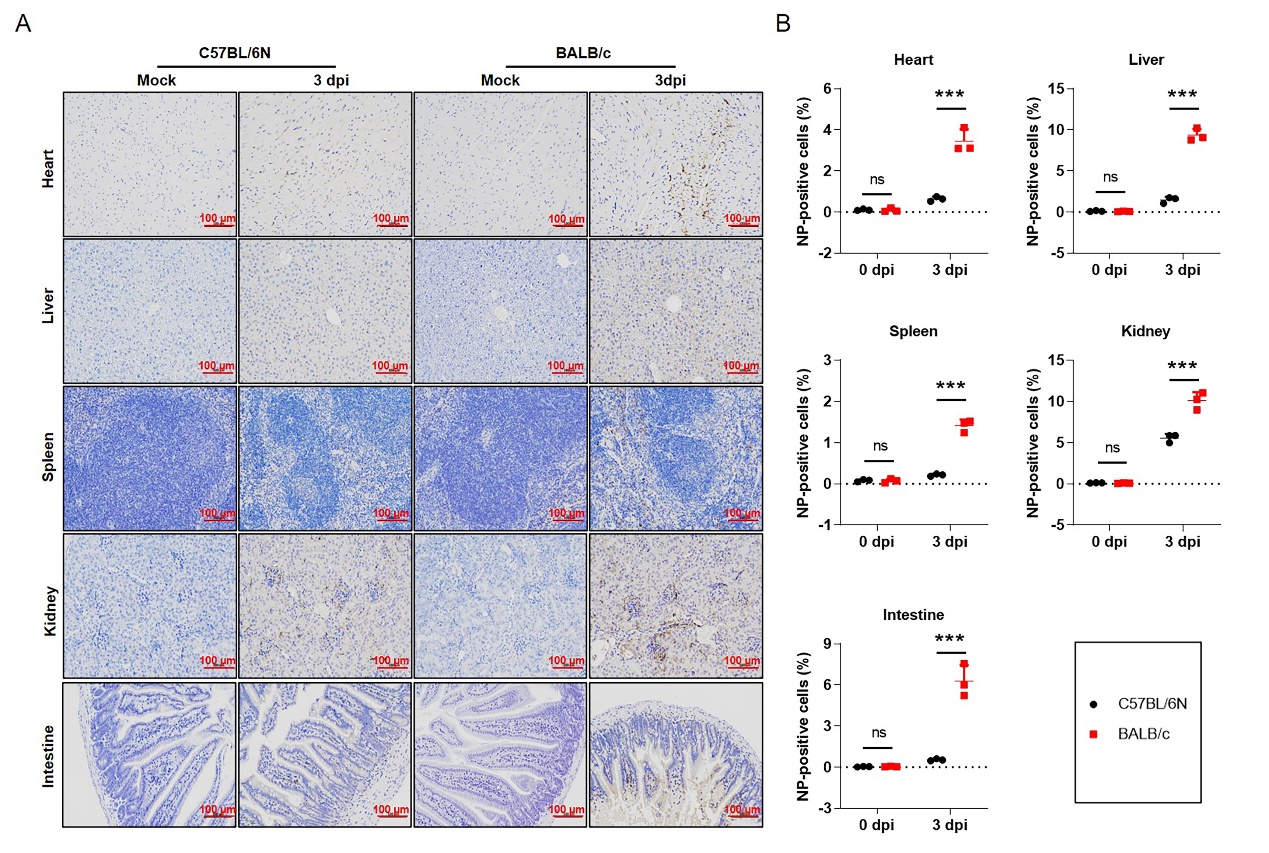


**Fig. S3 The NP expression in lung tissues of infected mice. (A)** IHC analysis of NP expression in tissues at 0 dpi and 3 dpi, showing representative photos of different groups, including heart, liver, spleen, kidney and intestinal tissues. **(B)** The percentage of NP-positive cells were summarized according to IHC staining in (A). ***p < 0.001, ns means no statistical difference.

**
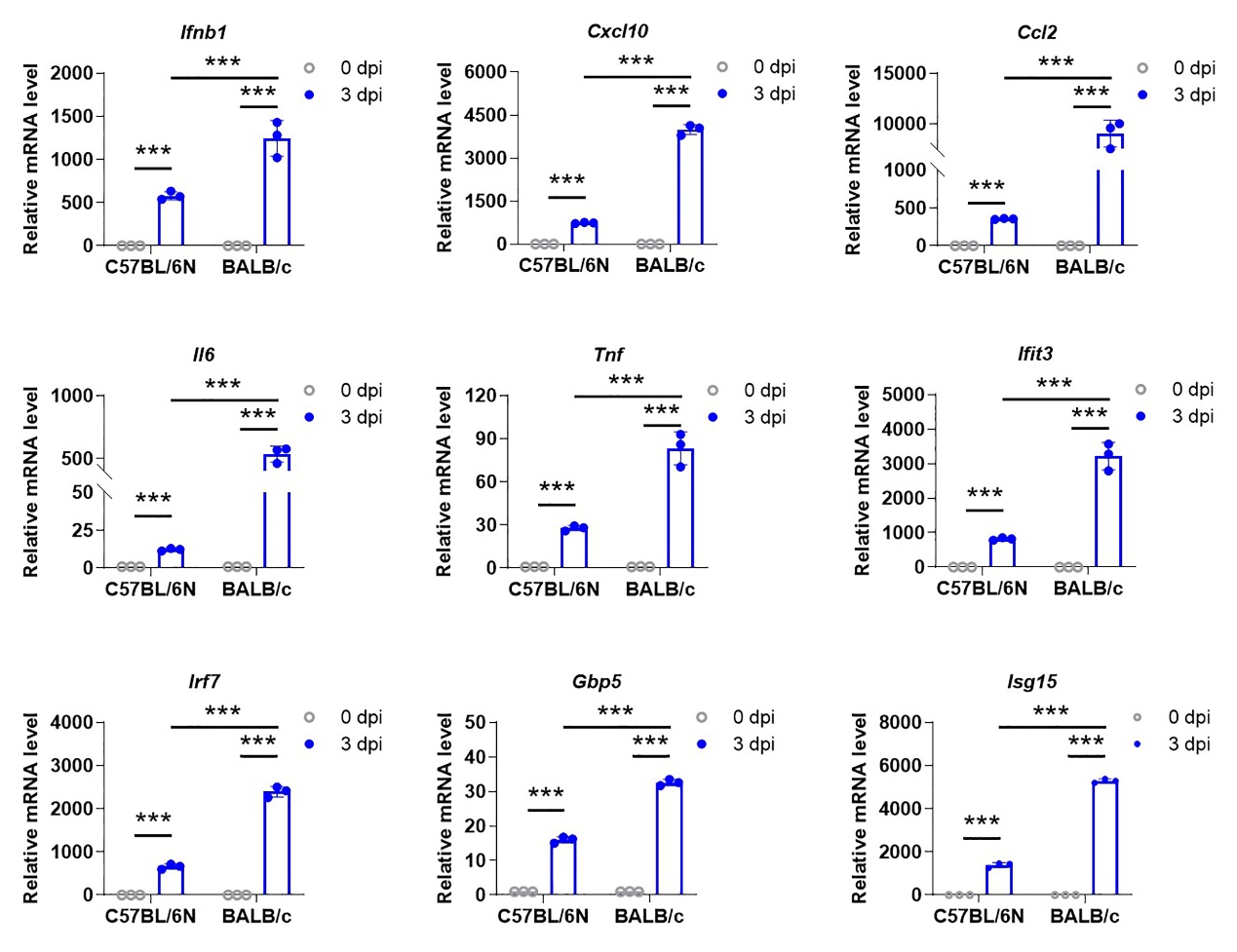
**

**Fig. S4** The effects of BMA8 strain infection on the expression of inflammation-related genes in lung tissues of C57BL/6N and BALB/c mice at 0 dpi and 3 dpi, including *Ifnb1*, *Cxcl10*, *Ccl2*, *Il6*, *Tnf*, *Ifit3*, *Irf7*, *Gbp5* and *Isg15.* ***p < 0.001.


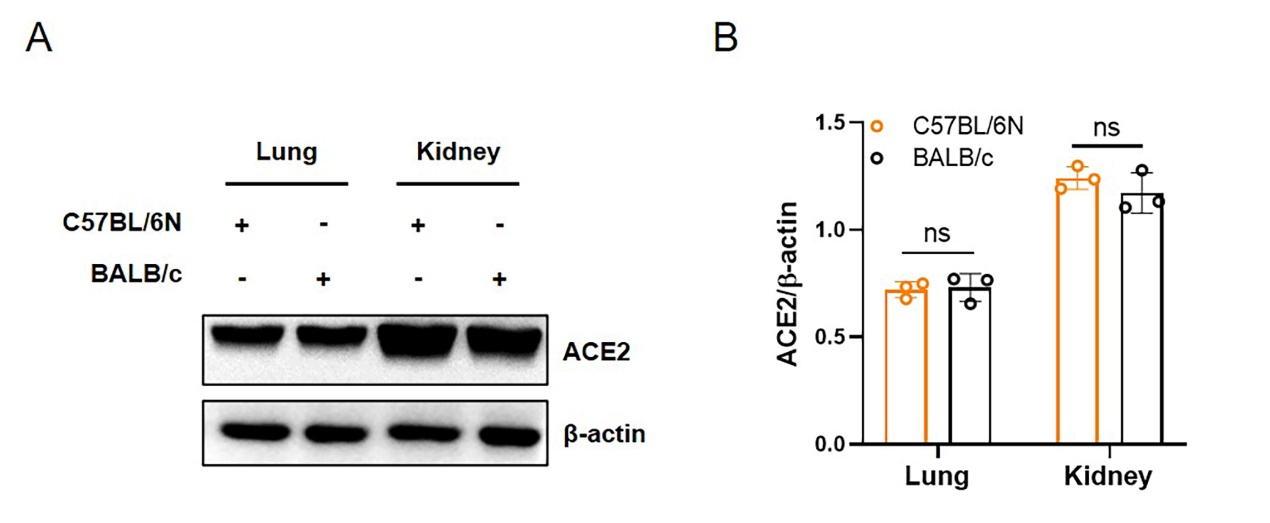


**Fig. S5** The expression levels of ACE2 protein in lung and kidney tissues of C57BL/6N and BALB/c mice; β-actin was used as a loading control. ns means no statistical difference.


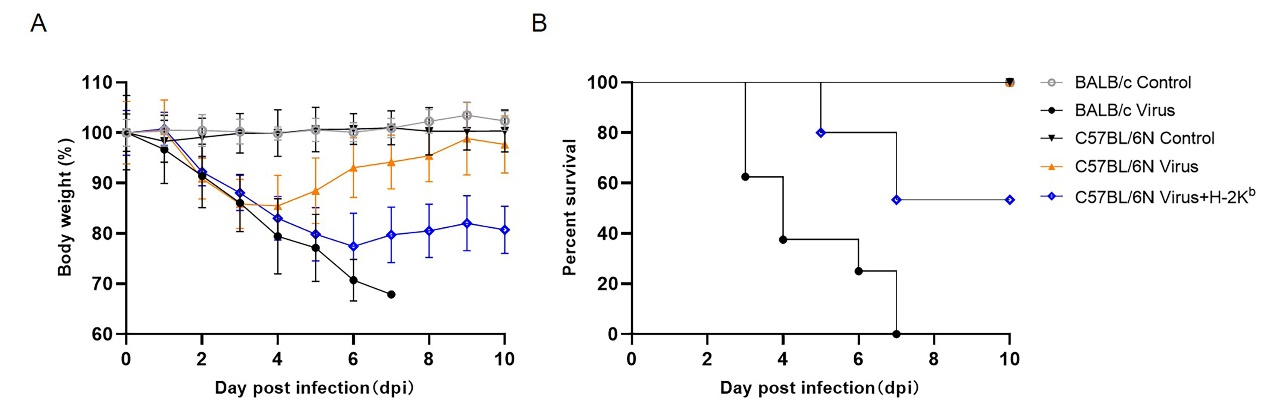


**Fig. S6** **Effects of H-2K^b^ mAbs on body weight and survival rate of mice infected with BMA8 strain. (A)** Analysis of body weight changes in mice. **(B)** Survival curve of mice, n=8.


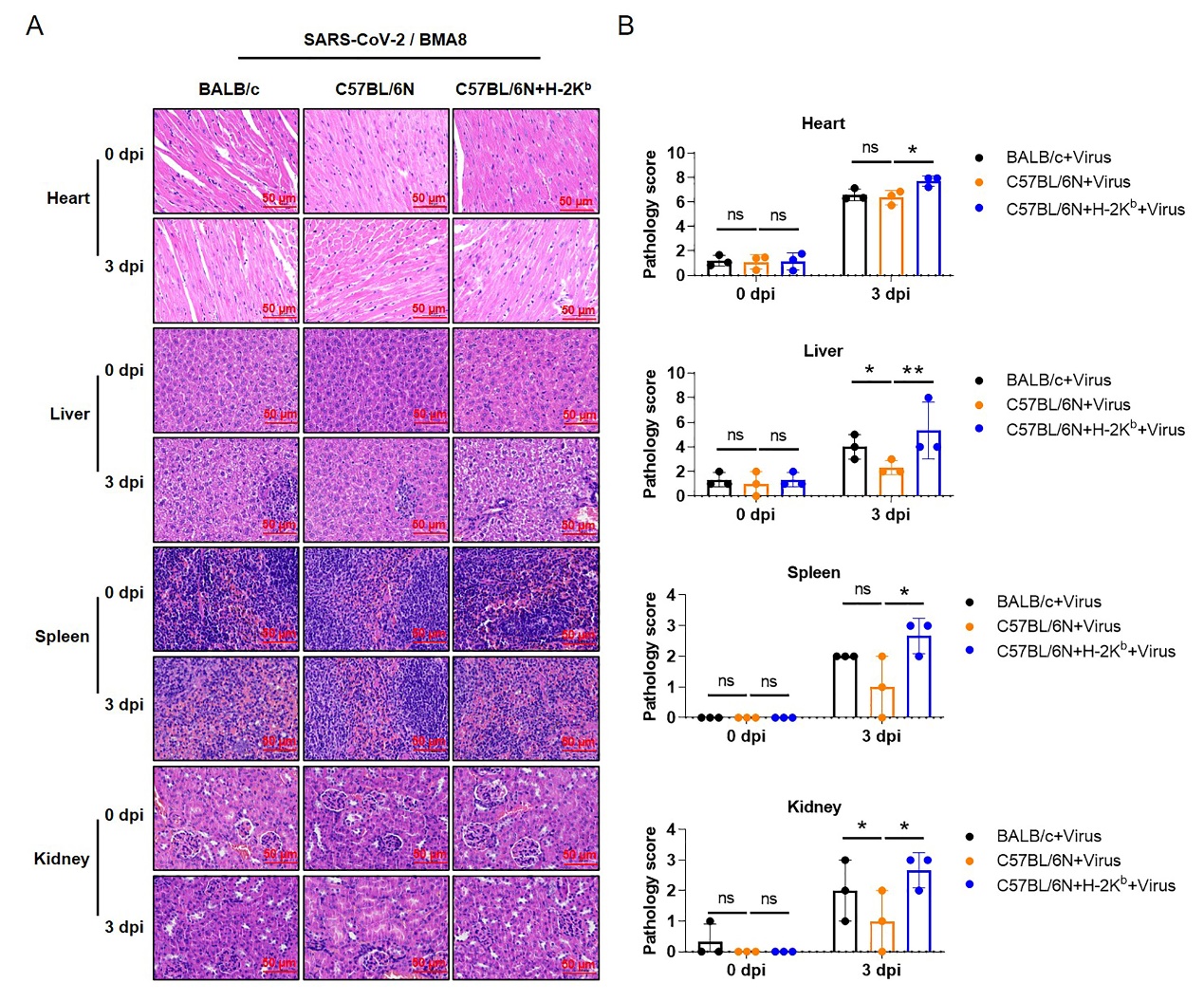


**Fig. S7 Effects of H-2K^b^ mAbs on pathogenicity of BMA8 strain in C57BL/6N mice.** **(A)** H&E staining was performed on the tissues of mice at 0 dpi and 3 dpi, showing representative photos of different groups, including heart, liver, spleen and kidney tissues. **(B)** The pathology scores were summarized according to Fig. 2B. *p < 0.05, **p < 0.01, ns means no statistical difference.


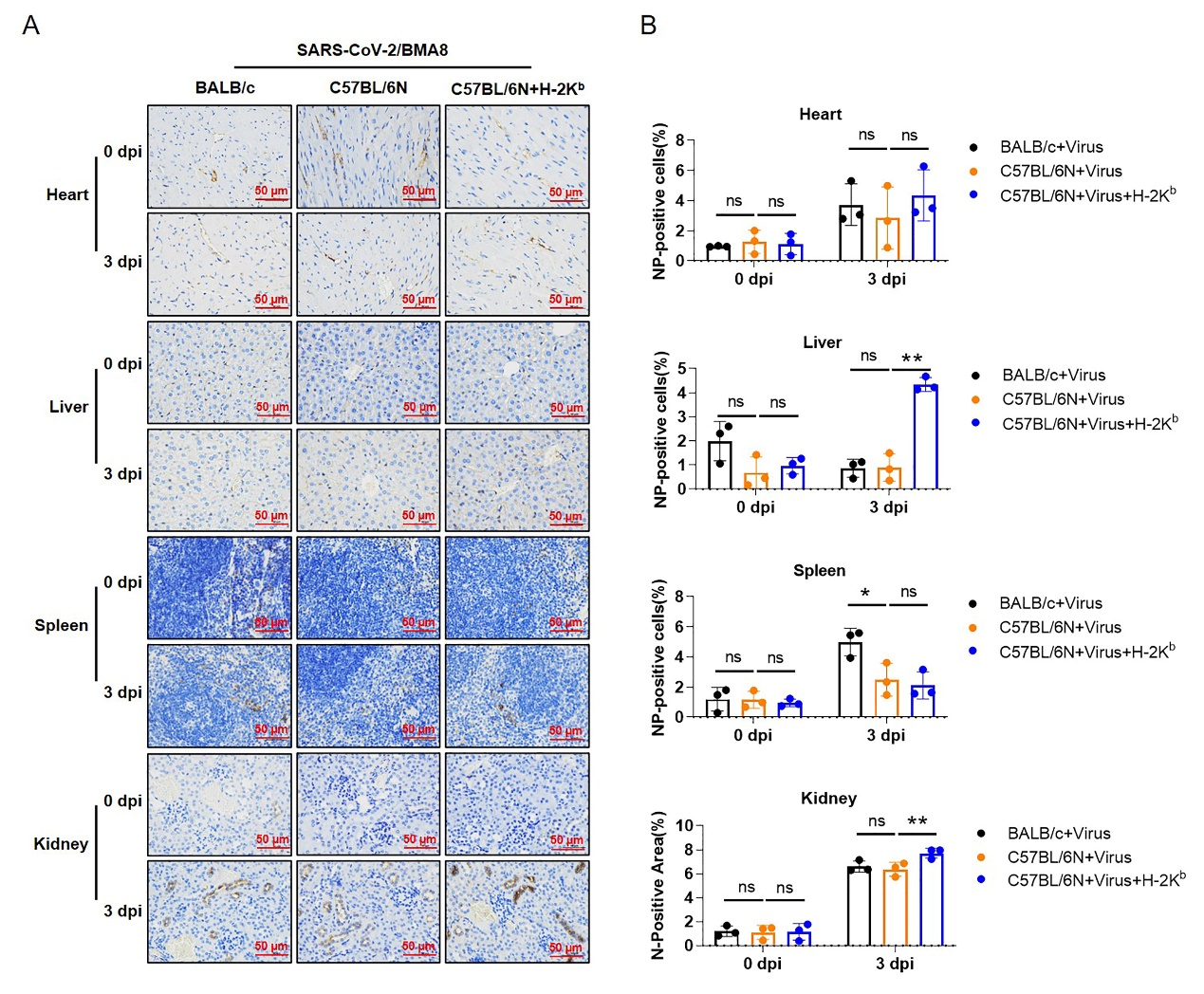


**Fig. S8 The expression of NP in lung tissues of infected mice.** **(A)** IHC analysis of NP expression in tissues at 0 dpi and 3 dpi with or without H-2K^b^ mAbs treatment, showing representative photos of different groups, including heart, liver, spleen and kidney tissues. **(B)** The percentage of NP-positive cells were summarized according to IHC staining in (A). *p < 0.05, **p < 0.01, ns means no statistical difference.


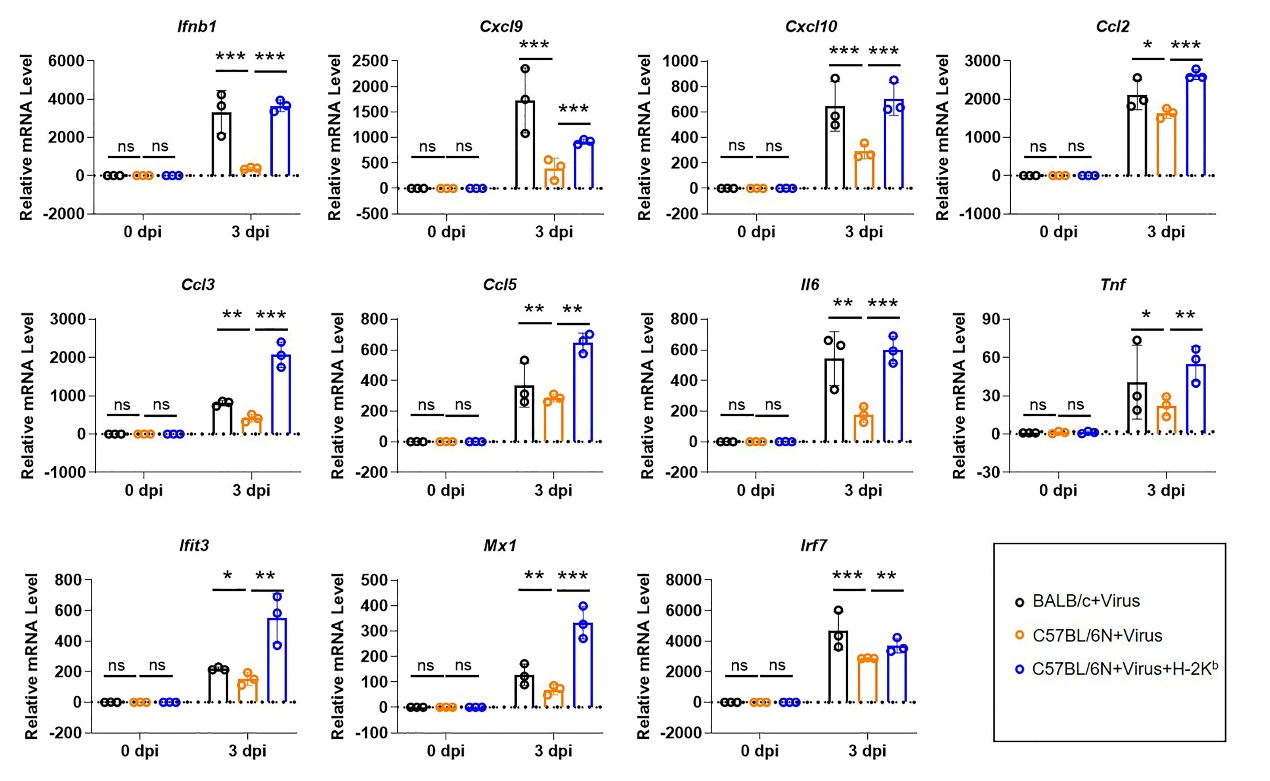


**Fig. S9** The effects of BMA8 strain infection on the expression of inflammation-related genes in lung tissues of C57BL/6N and BALB/c mice with or without H-2K^b^ mAbs treatment, including *Ifnb1*, *Cxcl9*, *Cxcl10*, *Ccl2*, *Ccl3*, *Ccl5*, *Il6*, *Tnf*, *Ifit3*, *Mx1* and *Irf7.* *p < 0.05, **p < 0.01, ***p < 0.001, ns means no statistical difference.
